# PREVENTING FALLS WITH PERTURBATION-BASED BALANCE TRAINING: HOW LARGE ARE THE HIP JOINT CONTACT LOADS, AND ARE THEY SAFE?

**DOI:** 10.1093/geroni/igac059.145

**Published:** 2022-12-20

**Authors:** David Graham, Justin Whitten, Bryant Oleary, Rod Barrett, Dawn Tarabochia

**Affiliations:** Montana State University, Bozeman, Montana, United States; Montana State University, BOZEMAN, Montana, United States; Montana State University, Bozeman, Montana, United States; Griffith University, Gold Coast, Queensland, Australia; Montana State University, Bozeman, Montana, United States

## Abstract

Perturbation-based training (PBT) is a balance training method presenting a high-challenge to balance which is extremely effective when compared to conventional training approaches. Common PBT methods use rapid treadmill belt translations with varying numbers of perturbations (20-1400 perturbations over 1-8wks). Importantly, the joint loads experienced during a stumble may be high enough to feasibly fracture bone (up to 12.7 Body Weights). However, the contact loads experienced during PBT are unknown. Because of increasing the prevalence of PBT it is necessary to specifically evaluate the range of joint loads experienced during PBT. Twelve participants completed a single PBT session of 24 perturbations. During both training and testing, participants movements were measured using a motion capture system which tracks body movements and records the forces under the feet. Hip joint contact loads were determined using Computational Musculoskeletal Modelling utilizing open-source software OpenSim. These techniques estimate the magnitude and pattern of force development of individual muscles and subsequently estimate the internal loads experienced by the hip joint.Hip joint contact loads were 4.90 ± 1.27 BW which is substantially lower that those previously reported by Graham et al. (2016) and Bergman et al. (2004) and is lower than the 5.5BW spontaneous fracture load boundary estimated by Schileo et al. (2014). Comparing the initial perturbation to the final perturbation revealed a 22% reduction in contact loads. We conclude that PBT performed using rapid translations on a treadmill are likely safe but suggest caution for individuals with poor bone mineral density or reduced neuromuscular function.

